# Focusing on Microenvironmental Disturbance to Potentiate Reparative Ability of Neural Stem Cell After Stroke

**DOI:** 10.34133/research.0829

**Published:** 2025-09-05

**Authors:** Hongfei Ge, Haomiao Wang, Rong Hu

**Affiliations:** Department of Neurosurgery, Southwest Hospital, Third Military Medical University (Army Medical University), 400038 Chongqing, China.

## Abstract

Ischemic stroke (IS), a common neurological disease, lacks satisfactory treatments worldwide. Neural stem cell (NSC) therapy is a promising strategy for stroke, while microenvironmental disturbance, including but not limited to acidosis, oxidative stress, and excessive neuroinflammation, restricts the therapeutic potential of endogenous and exogenous NSC post-IS. The present study introduces the effect of common disturbance on NSC and the underlying mechanism to screen out feasible methods aiming to potentiate the reparative capacity of NSC following IS.

## Introduction

**Fig. 1.**
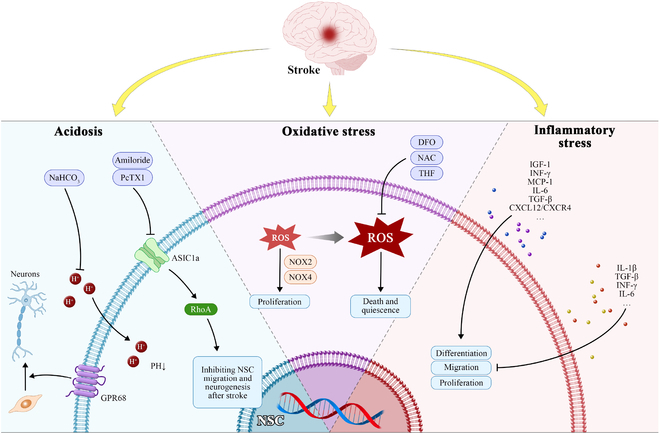
The underlying mechanisms of microenvironmental disturbance restricting NSC participating in regeneration and approaches to potentiating their reparative ability post-stroke. O_2_, oxygen; NOX2, NADPH oxidase 2; BDNF, brain-derived growth factor; SOD, superoxide dismutase; NAC, N-acetylcysteine; GPX, glutathione peroxidase; ASIC1a, acid-sensing ion channels 1a; GPR68, G protein-coupled receptor 68; IGF-1, insulin-like growth factor-1; EGF, epidermal growth factor; VEGF, vascular epidermal growth factor; bFGF, basic fibroblast growth factor; M1, M1-like microglia; M2, M2-like microglia.

Ischemic stroke (IS), a leading cause of death and severe disability worldwide, is clinically characterized as a sudden interruption of the brain blood flow resulting in neurological deficits of the central nervous system (CNS) [1]. Once IS occurs, endogenous neural stem cell (NSC) hold the ability of restoring damaged neural tissue via differentiation into neurons, astrocytes, and oligodendrocytes around lesions after stroke [2]. However, the reparative capacity of endogenous NSC is limited. Alternatively, exogenous NSC transplantation has emerged as a promising strategy for supplementing the insufficient number of endogenous NSC [3]. Unfortunately, neither endogenous nor exogenous NSC survive for a long period and poorly differentiate into functional neurons to repair damaged neural circuit. The main reason for this phenomenon might attribute to the microenvironmental disturbance that restricts the therapeutic potential of NSC after stroke [4]. This perspective highlights the effect of microenvironmental disturbance on NSC including endogenous and exogenous NSC, and provides new insights for enhancing reparative capacity of NSC in the treatment of IS ( Fig. 1).

## The Common Microenvironmental Disturbance After IS

### Low pH

Once stroke occurs, the increased intracranial pressure (ICP) caused by swelling and edema usually induces hypoperfusion of brain tissue, which results in a mildly acidic environment. Meanwhile, the switch from aerobic metabolism to anaerobic glycolysis induces the deposition of lactic acid and pyruvate that introduces decreased pH following IS [5]. Moreover, hydrogen ions deriving from the hydrolysis of excessive adenosine triphosphate exaggerate acidosis surrounding the ischemic core [6]. Additionally, the elevated tissue carbon dioxide pressure and spreading depolarization assist to exaggerate microenvironmental acidosis. The microenvironmental pH value is going to obviously drop from normal level to 6.5 in mild cases and even to 6.0 in severe cases to form microenvironmental acidification [7]. Low pH, also called acidosis, is a feather in the acute phase following IS.

### Increased oxidative stress

Oxidative stress deriving from the disequilibrium between depletion of antioxidants and production of reactive oxygen species (ROS) is a key hallmark of IS. The excessive deposition of ROS, such as superoxide (O_2_^−^·), hydrogen peroxide (H_2_O_2_), lipid peroxides (ROOH), or the corresponding hydroxyl (HO·) and peroxyl radicals (ROO·), usually initiates oxidative stress [8]. In the meantime, mitochondrial dysfunction resulting from metabolic transfer assists ROS accumulation and facilitates antioxidant depletion including superoxide dismutase (SOD), glutathione peroxidase (GPX), vitamins E and C, and N-acetylcysteine (NAC). The excessive ROS reacts with lipids, proteins, and DNA, leading to cell dysfunction, injury, and inflammation, ultimately inducing cell death [6]. Oxidative stress plays evident roles in different pathological stages after IS and gradually equilibrates over time.

### Neuroinflammation

Neuroinflammation persists and brings about individual pathology at different stages after IS. Following IS, apoptotic and necrotic cells release diverse danger-associated molecular patterns (DAMPs), which facilitate the accumulation of inflammatory chemokines and cytokines, such as tumor necrosis factor-α (TNF-α), monocyte chemotactic protein (MCP), interleukin-6 (IL-6), and interleukin-1β (IL-1β) [6]. These factors promote microglia transformation into M1 phenotype, which secrete pro-inflammatory mediators. Furthermore, the deposition of inflammatory mediators facilitates the infiltration of peripheral immune cells, such as neutrophils, monocytes, and macrophages around the infarct core [9]. The enrichment of peripheral immune cells further aggravates microvascular occlusion and exacerbates the release of DAMPs and ROS that initiates neuronal damage and extends a vicious cycle of neuroinflammation [6], while neuroinflammation also exerts beneficial effects by scavenging cellular debris and promoting tissue repair [10]. Herein, the role of neuroinflammation is complex and multifaceted after stroke.

Together, acidosis, oxidative stress, and aberrant inflammation are the common microenvironmental disturbance after IS. The activation of endogenous NSC and transplantation of exogenous NSC have proven to exert multiple beneficial effects through cell replacement and bystander effects (e.g., neuroprotection, immunomodulation, and vascularization) [11]. Hence, deciphering the effect and underlying mechanisms of microenvironmental oscillation on NSC (both endogenous and exogenous NSC) helps develop targeted therapies to boost the reparative capability of NSC for developing effective therapies to minimize secondary injury and ameliorate outcomes.

## The Effect and Mechanistic Insights of Microenvironmental Disturbance on Reparative Ability of NSC Following IS

Extreme microenvironmental disturbance will inevitably lead to NSC death, while moderate microenvironmental imbalance could not induce NSC loss, but restricts the reparative capacity of NSC after IS.

### Acidosis

When NSC are exposed to extracellular acidosis, their intracellular pH values become decreased as the cell membranes are permeable to protons [12]. Previous study certifies that NSCs express G protein-coupled receptor 68 (GPR68), a proton-sensitive receptor. NSC could sense extracellular niche change, and transient mild acidosis resulting from exercise promotes NSC localized at subgranular zone (SGZ) differentiation into neurons in hippocampus through GPR68 in adult mouse [13]. Most recently, our previous study demonstrates that acid-sensing ion channels 1a (ASIC1a), a subunit of proton-gated cation channels, is expressed in NSC. The activation of ASIC1a impairs the migration of NSC and neurogenesis in penumbra by eliciting downstream effector of RhoA signaling to trigger reorganization of the cytoskeleton and filopodia formation after IS [14]. Furthermore, the administration of ASIC1a antagonist or ASIC1a deletion promotes functional recovery by enhancing NSC migration toward penumbra and neurogenesis after IS [14].

### Oxidative stress

Excessive oxidative stress induces NSC death and quiescence, and produces immediate progenitor through various key effectors, such as NADPH (reduced form of nicotinamide adenine dinucleotide phosphate) oxidase 2 (NOX2) or NOX4, which maintains stemness and promotes NSC proliferation, thereafter improving the reparative ability of NSC in the injured brain. Meanwhile, elevated ROS levels enhance activation of different signaling to alter NSC migration and differentiation, dendrite development, and neuronal maturation including phosphatidylinositol 3-kinase (PI3K)/AKT, type I interferon, and Wnt/β-catenin [11]. Once oxidative stress occurs, antioxidant response is negatively activated to combat noxious effects of oxidative insults. These anti-oxidative stress responsive transcription factors are substantial for maintaining NSC reservoir and their function. For example, PR domain containing 16 (PRDM16) is required for NSC maintenance and mediation of NSC behavior. Nuclear factor erythroid 2-related factor 2 (Nrf2), a master regulator of the cellular defense against oxidative stress, is crucial for NSC function, whose knockout impairs NSC stemness and neuronal differentiation [15].

### Inflammatory stress

The local inflammatory stress is triggered by cytokines and chemokines that exert various functions on NSC after IS. For example, IL-1β inhibits NSC proliferation through nuclear receptors in the hippocampus. Interferon-γ (INF-γ) arrests NSC in G1 phase through signal transducer and activator of transcription (STAT1) and STAT3, thereafter reducing the ability of nerve regeneration by inhibiting NSC proliferation. INF-γ promotes the proliferation of cortical neural precursor cells (NPCs) by activating Shh. Therefore, the effect of INF-γ on NSCs relies on the activation of different signaling pathways. With the increase of transforming growth factor-β (TGF-β) expression, the proliferative activity of NSC is significantly reduced by arresting NSC in G0 phase of the cell cycle, but it promotes the survival of NPCs. Additionally, insulin-like growth factor-1 (IGF-1) promotes NSC proliferation through the extracellular signal-regulated kinase (ERK)/mitogen-activated protein kinase (MAPK) signaling pathway. The activation of the chemokine C-X-C motif ligand 12 (CXCL12)/C-X-C chemokine receptor type 4 (CXCR4) axis promotes neural regeneration by potentiating NSC migration toward the lesion and enhancing the blood–brain barrier (BBB) remodeling by elevating the expression of epidermal growth factor receptor (EGFR) and integrin α6, and integrating with vascular endothelial cells after brain injury. MCP-1 facilitates NSC recruitment into the injured site and participates in nerve regeneration in the chronic phase after stroke. IL-6 potentiates the differentiation of NSC into glial cells through STAT3 and Smad1 signaling pathways while inhibiting their differentiation into neurons.

Based on above evidence, NSC could sense and interact with common microenvironmental disturbance (acidosis, oxidative stress, and neuroinflammation). Herein, developing strategies targeting microenvironmental disturbance using specific methods at different stages is a feasible approach enhancing reparative ability of NSC, ultimately accelerating neurorehabilitation after stroke.

## Strategies to Enhancing the Reparative Capacity of NSC Based on Microenvironment Disturbance

### Endogenous NSC

Based on common microenvironmental disturbance, some strategies have been developed. The administration of ASIC1a blocker (amiloride or PcTX1) facilitates endogenous NSC migration and neurogenesis after IS [14]. ROS accumulation enhances NSC proliferation at the acute stage, but inhibits NSC proliferation and impairs neurogenesis in the subacute and chronic phase after stroke. The administration of deferoxamine (DFO) and NAC (a ROS scavenger) offsets this effect [16]. Furthermore, reducing ROS level using tetrahydrofolate (THF), an effective antioxidant, activates the PTEN/AKT/mammalian target of rapamycin (mTOR) pathway to potentiate the rehabilitative capacity of NSC in the treatment of CNS diseases with the presence of oxidative stress [17].

With the recruitment of NSC around infarct core, the endogenous NSC could exert versatile effects to mitigate microenvironmental imbalance. Firstly, the endogenous NSCs alleviate excessive neuroinflammation by inhibiting the activation of M1 microglia, which release pro-inflammatory factors such as IL-1β and INF-γ at the injured sites. With the decrease of pro-inflammatory factors, the excessive neuroinflammation is attenuated to promote in situ neural cells survival. Meanwhile, it facilitates NSC proliferation and differentiation into neurons. Furthermore, NSCs reinforce microglia polarization from M1 to M2 and release anti-inflammatory factors [18]. With the increase of anti-inflammatory elements, the excessive neuroinflammation is further alleviated, initiating a positive feedback loop to resisting the neuroinflammatory response. Secondly, the NSC secrete a series of neuroprotective factors, such as vascular endothelial growth factor (VEGF), epidermal growth factor (EGF), brain-derived neurotrophic factor (BDNF), and basic fibroblast growth factor (bFGF), to mitigate microenvironmental disturbance and enhance neurovascular remodeling. For example, autocrine VEGF by NSC facilitates paracrine VEGF deriving from host astrocytes. The increased concentration of VEGF, which promotes the reconstruction of the damaged vascular network, contributes to mitigating acidosis by vascularization, reducing ROS accumulation and pro-inflammatory factors to turn the “desert into an oasis” and to improve the local microenvironmental disturbance. NSCs obviously proliferate as EGF binding to its receptor EGFR. BDNF and nerve growth factor (NGF) are essential for maintaining the survival of NSCs and neurons, and promoting neuronal differentiation after brain development or injury.

### Exogenous NSC

Although various methods have been applied to potentiate the reparative capacity of endogenous NSC, the number of endogenous NSC is insufficient. Exogenous NSC transplantation is an effective way to supplement the insufficient number of endogenous NSC. However, the transplanted NSCs face the same problem as the endogenous NSCs that is the common microenvironmental imbalance. Herein, several approaches have been developed to promote NSC proliferation, migration, and proper differentiation, prior to implantation. First, gene transfection by incorporating specific gene into genome has been extensively performed to improve the therapeutic ability of engrafted NSC, such as VEGF enhancing vascularization, CXCR4 promoting NSC homing, and BDNF facilitating NSC differentiation into neurons. Second, inorganic nanoparticles have been developed to resist hypoxic injury by up-regulating antioxidant defense and inhibiting apoptosis using melanin nanoparticles. Fe_3_O_4_ nanoparticles combined with antioxidant layer are used to achieve a higher NSC survival rate against ROS microenvironment and long-term in vivo tracking using magnetic resonance imaging (MRI) [19]. Third, modifying the surface of living NSC with natural or synthetic biomaterials is an effective method to improve their survival rate and desired differentiation, such as NSCs modified by gelatin and hyaluronic acid (HA) tethered with VEGF using layer-by-layer (LbL) assembly [20]. Fourth, pretreatment with hypoxia, glial cell line-derived neurotrophic factor (GDNF), BDNF, IL-1β, TNF-α, and certain drugs promotes the secretion of VEGF, GDNF, and CXCR4 to enhance implanted NSC survival, homing, and differentiation into functional neurons [19]. Some challenges need to be achieved, such as increasing transfection efficiency while avoiding cytotoxicity, and reducing immunogenicity and cytotoxicity resulting from gene transfection reagents and nanoparticles.

## Concluding Remarks

NSC have exhibited versatile therapeutic effects in the treatment of IS. The presence of the common microenvironmental disruption, including but not limited to acidosis, oxidative stress, and excessive neuroinflammation, restricts the curative efficiency of NSC following IS. In order to acquire better outcomes using NSC in the treatment of IS, future research should focus on (a) investigating other microenvironmental disturbance limiting the reparative ability of NSC, (b) deciphering the underlying mechanism to screen out target aiming to reinforce the capacity of endogenous NSC, (c) exploiting novel modified strategies facilitating exogenous NSC survival, proliferation, migration, and proper differentiation, (d) optimizing therapeutic time window using endogenous and exogenous NSC in different phases (acute, subacute, and chromic), and (e) introducing 3-dimensional (3D) culture system and developing novel pretreatment methods. In the future, as more valuable interactions between microenvironmental disturbance and NSC are uncovered, it is surely to pave the way for enhancing reparative ability of endogenous and exogenous NSC, introducing better quality of life and therapeutic outcomes for IS patients.
